# Treatment of the Cutaneous Disease Produced by Certain Vegetable Poisons

**Published:** 1829

**Authors:** 


38. *Treatment of the Cutaneous disease produced by certain vegetable poisons.*—Dr. Dakin, of Columbus, N. Y. (American Journal of the Medical Sciences, No. 7) informs the profession, that he has uniformly cured the diseases produced by the exhalations of the different species of Rhus, or poison vine, sumach, and poison oak—

By blood-letting, cooling laxatives of neutral salts, and the following unguent as a local application:—℞. Cupri sulph. ʒi.; Precip. mer. rub. ʒi.; Tereb. ven. ʒiij.; Axung. porc. ℥i.—M. ft. ungt.

On the slightest admonition that the disease is contracted, I abrade the cuticle, or open the pimples, and apply a small quantity of the ointment, which occasions a slight smarting, but agreeable, compared with the tormenting sensations attendant on the affection. One or two applications with about twelve hours intervening between them, will arrest the inflammation, and in three or four days all marks of disease are generally obliterated. Venesection is rarely necessary, except the eyes, scrotum, or some important organ be concerned.—The above ointment is most efficient in arresting the progress of the complaint if applied the day of its appearance; and though neglected for two, three, or four days, will generally give prompt relief.

The affected parts should be kept cool, and the patient be ordered not to handle his body, without first washing his hands, after touching the sores, as he might thus unconsciously inoculate other parts.

We recollect that many years ago this affection attracted the attention of the late Professor Barton, who employed for its cure a solution of corrosive sublimate—deuto-chloride of mercury. Of the relative value of this application and that of Dr. Dakin we are unable to speak experimentally.

